# Cardiorespiratory fitness, hippocampal subfield morphology, and episodic memory in older adults

**DOI:** 10.3389/fnagi.2024.1466328

**Published:** 2024-12-19

**Authors:** Hayley S. Ripperger, Rebecca G. Reed, Chaeryon Kang, Alina Lesnovskaya, Sarah L. Aghjayan, Haiqing Huang, Lu Wan, Bradley P. Sutton, Lauren Oberlin, Audrey M. Collins, Jeffrey M. Burns, Eric D. Vidoni, Arthur F. Kramer, Edward McAuley, Charles H. Hillman, George A. Grove, John M. Jakicic, Kirk I. Erickson

**Affiliations:** ^1^Department of Psychology, University of Pittsburgh, Pittsburgh, PA, United States; ^2^Department of Psychiatry, University of Pittsburgh, Pittsburgh, PA, United States; ^3^Center for the Neural Basis of Cognition, University of Pittsburgh and Carnegie Mellon University, Pittsburgh, PA, United States; ^4^Department of Neuroscience, AdventHealth, AdventHealth Research Institute, Orlando, FL, United States; ^5^The Grainger College of Engineering, Bioengineering Department, University of Illinois, Champaign, IL, United States; ^6^Beckman Institute, University of Illinois, Urbana, IL, United States; ^7^Weill Cornell Institute of Geriatric Psychiatry, Weill Cornell Medicine, White Plains, NY, United States; ^8^Department of Neurology, University of Kansas Medical Center, Kansas, KS, United States; ^9^Center for Cognitive and Brain Health, Northeastern University, Boston, MA, United States; ^10^Department of Psychology, Northeastern University, Boston, MA, United States; ^11^Department of Health and Kinesiology, University of Illinois, Urbana, IL, United States; ^12^Department of Physical Therapy, Movement, and Rehabilitation Sciences, Northeastern University, Boston, MA, United States; ^13^Department of Internal Medicine, University of Kansas Medical Center, Kansas, KS, United States

**Keywords:** hippocampus, cardiorespiratory fitness, hippocampal subfields, episodic memory, MRI, ASHS, brain aging

## Abstract

**Objective:**

Age-related hippocampal atrophy is associated with memory loss in older adults, and certain hippocampal subfields are more vulnerable to age-related atrophy than others. Cardiorespiratory fitness (CRF) may be an important protective factor for preserving hippocampal volume, but little is known about how CRF relates to the volume of specific hippocampal subfields, and whether associations between CRF and hippocampal subfield volumes are related to episodic memory performance. To address these gaps, the current study evaluates the associations among baseline CRF, hippocampal subfield volumes, and episodic memory performance in cognitively unimpaired older adults from the Investigating Gains in Neurocognition Trial of Exercise (IGNITE) (NCT02875301).

**Methods:**

Participants (*N* = 601, ages 65–80, 72% female) completed assessments including a graded exercise test measuring peak oxygen comsumption (VO_2peak_) to assess CRF, cognitive testing, and high-resolution magnetic resonance imaging of the hippocampus processed with Automated Segmentation of Hippocampal Subfields (ASHS). Separate linear regression models examined whether CRF was associated with hippocampal subfield volumes and whether those assocations were moderated by age or sex. Mediation models examined whether hippocampal volumes statistically mediated the relationship between CRF and episodic memory performance. Covariates included age, sex, years of education, body mass index, estimated intracranial volume, and study site.

**Results:**

Higher CRF was significantly associated with greater total left (*B* = 5.82, *p* = 0.039) and total right (*B* = 7.64, *p* = 0.006) hippocampal volume, as well as greater left CA2 (*B* = 0.14, *p* = 0.022) and dentate gyrus (DG; *B* = 2.34, *p* = 0.031) volume, and greater right CA1 (*B* = 3.99, *p* = 0.011), CA2 (*B* = 0.15, *p* = 0.002), and subiculum (*B* = 1.56, *p* = 0.004) volume. Sex significantly moderated left DG volume (*B* = −4.26, *p* = 0.017), such that the association was positive and significant only for males. Total left hippocampal volume [indirect effect = 0.002, 95% CI (0.0002, 0.00), *p* = 0.027] and right subiculum volume [indirect effect = 0.002, 95% CI (0.0007, 0.01), *p* = 0.006] statistically mediated the relationship between CRF and episodic memory performance.

**Discussion:**

While higher CRF was significantly associated with greater total hippocampal volume, CRF was not associated with all underlying subfield volumes. Our results further demonstrate the relevance of the associations between CRF and hippocampal volume for episodic memory performance. Finally, our results suggest that the regionally-specific effects of aging and Alzheimer’s disease on hippocampal subfields could be mitigated by maintaining higher CRF in older adulthood.

## Introduction

Aging is associated with progressive decline in the size of the hippocampus, a medial temporal lobe structure supporting episodic and relational memory processes (Burgess et al., 2002). Normative aging is associated with 1–2% annual decline in the size of the hippocampus starting around age 50–55 (Raz et al., 2005), while individuals with mild cognitive impairment (MCI) or Alzheimer’s disease (AD) demonstrate more precipitous rates of deterioration (Jack et al., 1998). This decline in total hippocampal volume is related to the age-related decline in episodic memory, or memory of past events and experiences (Tulving, 2002; Head et al., 2008). Yet, the hippocampus is not a homogenous structure; it is made up of several primary subfields (CA1-4, dentate gyrus (DG), and subiculum), which have distinct structural and functional characteristics (Zeidman and Maguire, 2016; Paxinos and Mai, 2004).

Hippocampal subfields differentially support episodic memory processes (Dimsdale-Zucker et al., 2018; Kesner and Rolls, 2015). Evidence from both animals and humans suggests that the subfields play distinct but complementary roles in episodic memory. For example, CA3/DG supports pattern separation (Jonas and Lisman, 2014; Yassa et al., 2011) and memory encoding (Hainmueller and Bartos, 2020; Hrybouski et al., 2019), whereas CA1 supports pattern completion (Lacy et al., 2011) and autobiographical memory retrieval (Bartsch et al., 2011). The subiculum has a number of functions but may be particularly important for spatial memory (de Melo et al., 2023; O’Mara et al., 2009). Notably, these subfields undergo aging- and AD-related atrophy at different rates (Small et al., 2011; Adler et al., 2018; Nadal et al., 2020). For example, the CA1 and subiculum subfields shrink disproportionately in individuals with MCI and early AD, and this distinctive subfield atrophy pattern is associated with later conversion from MCI to AD (de Flores, De Flores et al., 2015).

Age-related hippocampal atrophy and its consequences for memory performance have led to a search for measures and approaches that could predict individual variability in hippocampal atrophy, as well as mitigate the rate of decline. One such factor is cardiorespiratory fitness (CRF), a physiological measure of aerobic capacity that influences risk for age-related cognitive decline and dementia (Tari et al., 2019), is associated with better episodic memory performance (Rigdon and Loprinzi, 2019; Hayes et al., 2014), and is modifiable by regular participation in aerobic physical activity (Lin et al., 2015). There have been a number of studies indicating that higher CRF is associated with larger total hippocampal volumes in older adults (Bugg et al., 2012; Erickson et al., 2009; Fletcher et al., 2016; McAuley et al., 2011; Szabo et al., 2011), but there have been others that have failed to detect statistically significant associations (Cole et al., 2020; Dougherty et al., 2017; Engeroff et al., 2018; Honea et al., 2009; Niemann et al., 2014; Raichlen et al., 2020).

One possible reason for the heterogenous findings is that these prior studies have used approaches that focus on total hippocampal volume rather than subfield volumes. If some subfields are more sensitive to CRF than others, then aggregating across subfields to measure total hippocampal volume may mask significant associations between CRF and particular subfields. Results from rodent studies support the argument that the effects of aerobic exercise on hippocampal morphology are subfield-specific, with the most consistent evidence for effects on the volume of the DG via increased neurogenesis (Biedermann et al., 2016; Farmer et al., 2004; Pereira et al., 2007), although the limited human literature on this is less consistent (Rosano et al., 2017; Rehfeld et al., 2017; Pani et al., 2021; Sakhare et al., 2021). To our knowledge, there has been only one other study in healthy older adults that examined the association between CRF and subfield volumes. Kern and colleagues demonstrated that in their sample (n = 34), higher CRF was associated with larger bilateral subiculum volume in females but not males (Kern et al., 2021).

In addition to subfield specificity, other factors such as sex and age might also explain heterogeneity within and between studies. Sex differences might modify the relationship between CRF and hippocampal volume metrics, possibly due to differences in glucometabolic and hypothalamic–pituitary–adrenal axis response to aerobic exercise (Baker et al., 2010), as well as the impact of changes in sex hormones in postmenopausal females, (Braden et al., 2017; Erickson et al., 2005; Rehbein et al., 2021). Age might also moderate the associations between CRF and hippocampal volume. For example, one study found that age moderated the association between CRF and tissue density (Colcombe et al., 2003), suggesting that higher CRF might become more important for mitigating brain atrophy among the oldest older adults.

Collectively the evidence we have reviewed indicates that although there are known associations among age-related hippocampal atrophy, memory performance, and aerobic exercise or CRF, there is a significant gap in the literature regarding whether these findings are driven by underlying hippocampal subfields, and whether such associations may depend on sex or age. To address these gaps in the literature, we examined associations among CRF, hippocampal total and subfield volumes, and episodic memory in a large and well-characterized sample of cognitively normal older adults. We hypothesized that higher CRF would be associated with larger total hippocampal volumes, and that the associations with underlying subfield volumes would be regionally-specific, such that CRF would be associated with the volume of some subfields but not all. In addition, we hypothesized that associations between hippocampal subfield volumes and CRF would be moderated by both age and sex, such that the associations would be weaker in younger ages and in males. Lastly we hypothesized that the association between CRF and episodic memory would be mediated by hippocampal subfield volume.

## Methods

### Participants and procedure

Participants were cognitively unimpaired older adults who were enrolled in a randomized controlled trial of aerobic exercise (Investigating Gains in Neurocognition in an Intervention Trial of Exercise (IGNITE): NCT02875301, R01AG053952). Participants were enrolled on a rolling basis between 2017 and 2022 with recruitment of racially and ethnically underrepresented participants proportional to the demographic characteristics of each study site (Boston; Kansas City; Pittsburgh). Participants were eligible if they were 65–80 years old, able to walk without being limited by pain or use of walking devices, inactive (i.e., self-reported exercise that was less than 20 min, 3 days per week, of structured moderate-to-vigorous exercise over the last 6 months), living in the community, able to undergo an MRI, and have no diagnosis of a neurological disease. We performed comprehensive neuropsychological testing with consensus adjudication to exclude individuals with MCI and dementia, but given known limitations of neuropsychological testing and evaluation and the often variable definitions of MCI, it is possible that there were participants that were included that could be near the MCI range.

Participants were excluded if they met any of the following criteria: current diagnosis or treatment of a psychological disorder (e.g., clinical depression); history of major psychiatric illness (e.g., schizophrenia); current treatment for cancer; neurological condition or brain injury; Type I diabetes or uncontrolled Type II diabetes; alcohol or substance abuse within the last 5 years; current treatment for congestive heart failure, angina, uncontrolled arrhythmia, DVT, or other cardiovascular event; myocardial infarction, coronary artery bypass grafting, angioplasty, or other cardiac condition in the past year; regular use of an assisted walking device; MRI contraindications; not fluent in English; not medically cleared by primary care physician; engaging in >20 min on 3 days or more of structured moderate-to-vigorous exercise per week [see Erickson et al. (2019)] for a detailed description of the eligibility criteria. The study was approved by the Institutional Review Board at each site (Pittsburgh: STUDY19110244; Kansas: STUDY00140896; Northeastern: 17–05-02) and all participants provided written informed consent before data collection.

Data for this analysis focus on baseline measurements of CRF, MRI, and cognitive testing, which were conducted on separate days within a maximum of 8 weeks of each other. Of the 648 participants who were enrolled and randomized, 641 completed a baseline MRI. Of the 641 with MRI data, 39 were excluded due to improper placement of the field of view for the T2-weighted MRI sequence, which prevented accurate hippocampal segmentation, and 1 was excluded due to an incidental finding in the hippocampal region. This resulted in 601 participants with usable MRI data for analysis.

### Cardiorespiratory fitness

Participants completed a maximal graded exercise test to assess aerobic capacity following a modified Balke protocol (Balke and Ware, 1959). After a brief warm-up session, including measurement of resting blood-pressure and resting electrocardiogram (ECG) review, the participant walked on a motor-driven treadmill at a pace between 1.5–3.5 mph that resulted in a heart rate of approximately 70% of age-predicted maximal heart rate (APMHR), or a Rating of Perceived Exertion (RPE) of 11 on the Borg rating scale (Borg, 1982). Once the walking speed was chosen, the participant walked at that constant speed for the duration of the test with an incrementally increasing incline. The incline was increased in two-minute stages with a 2% increase in incline at each stage. Heart rate was continuously monitored via a 12 lead ECG along with blood pressure and RPE measured during the second minute of every stage. Oxygen consumption (VO_2_) was measured via exhaled air analyzed by metabolic carts (Parvo Medics TrueOne 2,400; COSMED Quark CPET) throughout the test. The highest level of VO_2_ (VO_2_peak) expressed relative to body weight (ml/kg/min) was used to represent CRF. The test was completed to volitional exhaustion or with symptom limitation. At test completion, a four-minute active cooldown was completed and followed by a four-minute resting cooldown. We also recorded whether the test met three of the four standard American College of Sports Medicine (ACSM) criteria for determining maximal CRF (American College of Sports Medicine, 2021): (1) Plateau in VO_2_ between two or more workloads (increase <0.15 L/min or 2.0 mL/kg/min during the last minute of corresponding workloads), (2) Respiratory Exchange Ratio (RER) ≥1.10, (3) Heart rate within 10 beats of the APMHR (220-age), and (4) an RPE ≥17. Maximal effort is typically defined as achieving at least 3 out of these 4 criteria. The majority of participants in the study met the 3 out of 4 criteria (see Table 1). These procedures have been previously described (Erickson et al., 2019; Oberlin et al., in press).

**Table 1:** Sample characteristics.

Measure	*N* = 601
Age (mean, SD)	69.7 (3.7)
Sex (*n*, %)	
Male	167 (27.8)
Female	434 (72.2)
Race (*n*, %)	
Caucasian/White	459 (76.4)
African American/Black	113 (18.8)
Bi-racial	10 (1.7)
Other	9 (1.5)
Asian	9 (1.5)
Native Hawaiian or other Pacific Islander	1 (0.2)
Years of Education (mean, SD)	16.3 (2.3)
BMI (kg/m^2^) (mean, SD)	29.8 (5.7)
CRF (VO_2peak_, ml/kg/min) (mean, SD)	21.7 (5.1)
CRF testing criteria (% to reach ¾)	80
Plateau in VO_2_ (%)	90
Max RER ≥ 1.1 (%)	47
Max RPE ≥ 17 (%)	90
HR within 10 of APMHR (%)	80

BMI, body mass index; CRF, cardiorespiratory fitness; RER, respiratory exchange ratio; RPE, rating of perceived exertion; HR, heart rate; APMHR, age-predicted maximal heart rate.

### Episodic memory composite

All participants completed a comprehensive neuropsychological battery during their baseline assessments. A confirmatory factor analysis (CFA) was conducted to obtain latent factors reflecting five core cognitive domains: episodic memory, processing speed, working memory, executive function/attentional control, and a visuospatial factor. The details and results from this analysis have been described in a prior publication (Oberlin et al., in press). In short, the CFA demonstrated good model fit (*χ*^2^ = 685.99, df = 259, *p* < 0.001, *χ*^2^/df = 2.649, CFI = 0.945, TLI = 0.936, RMSEA = 0.05, SRMR = 0.05), and all factor loadings were statistically significant (*p* < 0.001). Within the episodic memory factor, factor loadings of tests ranged from 0.55–0.74. The episodic memory composite factor included outcomes from the Brief Visuospatial Memory Test – Revised (BVMT) (Benedict et al., 1996), Hopkins Verbal Learning Test (HVLT) (Brandt, 1991), Picture Sequence (Dikmen et al., 2014), MoCA delayed recall (Nasreddine et al., 2005), Paired Associates (Salthouse et al., 1996), and Logical Memory (Tulsky et al., 2003), and the summary statistics for each of these are reported in Table 2.

**Table 2:** Cognitive tasks and outcomes for the episodic memory composite.

Cognitive task	Outcomes	Range	Mean (SD)
Logical memory (VCAP)	Immediate recall total score	10–66	43.43 (9.03)
	Delay recall total score	0–48	27.44 (7.01)
Paired associates (VCAP)	Immediate recall mean score	0–6	2.12 (1.41)
	Delay recall mean score	0–6	1.43 (1.39)
MoCA delayed recall	Delayed free recall	0–5	3.02 (1.55)
Picture sequence	Total raw score	0–31	10.37 (5.93)
Hopkins verbal learning test (HVLT)	Total immediate recall raw score	12–36	26.00 (4.49)
	Delayed recall (trial 4) raw score	1–12	9.15 (2.11)
	Recognition discrimination index score	4–12	10.62 (1.44)
Brief visuospatial memory test - revised (BVMT)	Total immediate recall raw score	3–36	21.10 (6.42)
	Delayed recall raw score	1–12	8.66 (2.53)

Range column depicts the lowest and highest scores obtained by participants on each cognitive task.

VCAP, version of the test that was adapted by Salthouse and colleagues as part of the Virginia cognitive aging project; MoCA, montreal cognitive assessment.

### MRI acquisition and hippocampal segmentation

Magnetic resonance images were collected on a Siemens Prisma 3 T scanner with a 64-channel head coil at two of the sites (University of Pittsburgh; Northeastern University) and on a Siemens Skyra 3 T scanner with a 32-channel head coil at the University of Kansas Medical Center. Images from a high-resolution T1-weighted 3D MPRAGE (Magnetization Prepared Rapid Gradient Echo Imaging) sequence (0.8 × 0.8 × 0.8 mm voxels, 224 slices, 0.8 mm slice thickness, TR = 2400.0 ms, TE = 2.31 ms, flip angle = 8 degrees) and T2-weighted focal hippocampal sequence [0.4 × 0.4 × 2.0 mm voxels, 30 slices, 2.0 mm slice thickness, TR = 8830.0 ms, TE = 78 ms, flip angle = 150 degrees, turbo spin echo (TSE)] were collected for hippocampal subfield segmentation. All MRI sequences across all sites were standardized and monitored for quality assurance on a weekly basis for the duration of the study.

To segment the subfields of the hippocampus, we used the Automatic Segmentation of Hippocampal Subfields (ASHS) software package, which uses multi-atlas segmentation and machine learning techniques to identify and label the subfields of the hippocampus and medial temporal lobe cortices (Yushkevich et al., 2015). ASHS requires a T1- and T2-weighted image and automatically labels the main hippocampal subfields on each participant’s T2-weighted image based on the Penn Memory Center 3 T ASHS atlas. For each participant, we used ASHS to generate measures of the subfields that make up the hippocampus proper: left and right CA1, left and right CA2, left and right CA3, left and right DG, and left and right subiculum. Of note, ASHS includes the hilus—which some consider a separate subfield, CA4—as part of the DG subfield. We then calculated measures of total left and right hippocampal volume by adding the subfields together. Each participant’s hippocampal segmentation results were visually examined to ensure that no major errors occurred during segmentation.

Rather than adjust each of our hippocampal variables for intracranial volume, we covaried for estimated intracranial volume in our statistical models. Estimated intracranial volume was calculated with FreeSurfer (version 6.0), which uses an atlas-based spatial normalization procedure to estimate the size of the intracranial vault (Buckner et al., 2004).

### Covariates

Covariates included age, sex, years of education, body mass index (BMI), estimated intracranial volume, and study site. Age, sex, and years of formal education were self-reported. Sex was either male or female, and no participants in the study identified as transgender or nonbinary. BMI (kg/m^2^) was calculated with height and weight collected using a calibrated stadiometer and scale at each site. Estimated intracranial volume was calculated with FreeSurfer (version 6.0) (Buckner et al., 2004). Study site (Pittsburgh, Kansas, or Northeastern) was included to account for potential site differences.

### Statistical analyses

All statistical analyses were conducted in R Studio (version 1.2.1335). Descriptive statistics were calculated, and CRF, hippocampal volume, and episodic memory variables were tested for normality of distribution. Independent samples t-tests (for continuous variables) and chi-square tests (for categorical variables) were conducted to assess whether there were significant differences between males and females on any demographic characteristics.

For Aim 1, we tested separate linear regression models to examine the association between CRF and total hippocampal volume, CA1 volume, CA2 volume, CA3 volume, DG volume, and subiculum volume.

For Aim 2, we tested whether any associations between CRF and hippocampal volumes were statistically moderated by age (entered as a continuous variable) or sex (entered as a binary variable) by testing CRF × age and CRF × sex interaction terms in separate regression models for each hippocampal variable. Simple slopes were calculated for all statistically significant interactions. Interactions with age as the continuous moderator were probed at one standard deviation (SD) units above and below the mean.

For Aim 3, we first tested whether CRF was associated with episodic memory performance using a linear regression model. We then tested separate mediation models to examine whether the association between CRF and episodic memory performance was mediated by the hippocampal volume variables. Statistical significance of the indirect paths was assessed by bootstrapping (5,000 iterations), with 95% confidence intervals (CIs) generated using the bias-corrected and accelerated method in the Causal Mediation Analysis package in R (Tingley et al., 2014).

Age, sex, BMI, education, and study site were included as covariates in all models. Intracranial volume was included as a covariate in all models examining hippocampal volume. Left and right hippocampal volume variables were examined separately (e.g., separate variables for left CA1 and right CA1). To adjust for multiple comparisons, we used the Benjamini-Hochberg correction with a false discovery rate (FDR) of 0.20 which was selected due to the novel and relatively exploratory nature of these analyses (McDonald, 2014). Results that survive FDR correction for multiple comparisons are reported as unstandardized coefficients (*B*) with uncorrected *p*-values. Standardized coefficients (*β*) are reported alongside the unstandardized results in Table 3.

**Table 3:** Associations between CRF and hippocampal volumes.

Outcome	*B* (*β*)	SE	*t*	*p*
*Predictor: CRF*				
Total left	5.82 (0.10)	2.80	2.07	0.039*
Left CA1	2.63 (0.08)	1.60	1.65	0.100
Left CA2	0.14 (0.13)	0.06	2.30	0.022*
Left CA3	0.11 (0.03)	0.17	0.65	0.514
Left DG	2.34 (0.12)	1.09	2.16	0.031*
Left subiculum	0.58 (0.05)	0.59	0.98	0.326
Total right	7.64 (0.14)	2.77	2.76	0.006*
Right CA1	3.99 (0.13)	1.57	2.55	0.011*
Right CA2	0.15 (0.17)	0.05	3.05	0.002*
Right CA3	0.12 (0.00)	0.20	0.59	0.557
Right DG	1.82 (0.09)	1.08	1.68	0.094
Right subiculum	1.56 (0.14)	0.54	2.90	0.004*

Unstandardized (*B*) and standardized (*β*) regression coefficients reflect the effect of CRF on each outcome variable. Hippocampal volume variables are measured in mm^3^, and CRF is VO_2peak_ in ml/kg/min.

*Statistically significant at uncorrected *p* < 0.05 and FDR < 0.2.

## Results

### Participants

Means and standard deviations of demographic variables and cardiorespiratory fitness are presented in Table 1. The 601 participants were, on average, 69.7 years old (± 3.7), female (72%), White (76%), with 16.3 years (± 2.2) of education. Average BMI would classify the sample as being overweight (29.8 ± 5.7), and average CRF (i.e., VO_2peak_) would indicate that our participants were generally low-fit (21.7 ± 5.1) (American College of Sports Medicine, 2021), although CRF was normally distributed and ranged from 10.1–39.6 mL/kg/min. The females in this sample were slightly younger, less educated, less fit, and more racially diverse than the males (see Supplementary Table 1).

### Associations between CRF and hippocampal volumes

**Figure 1:**
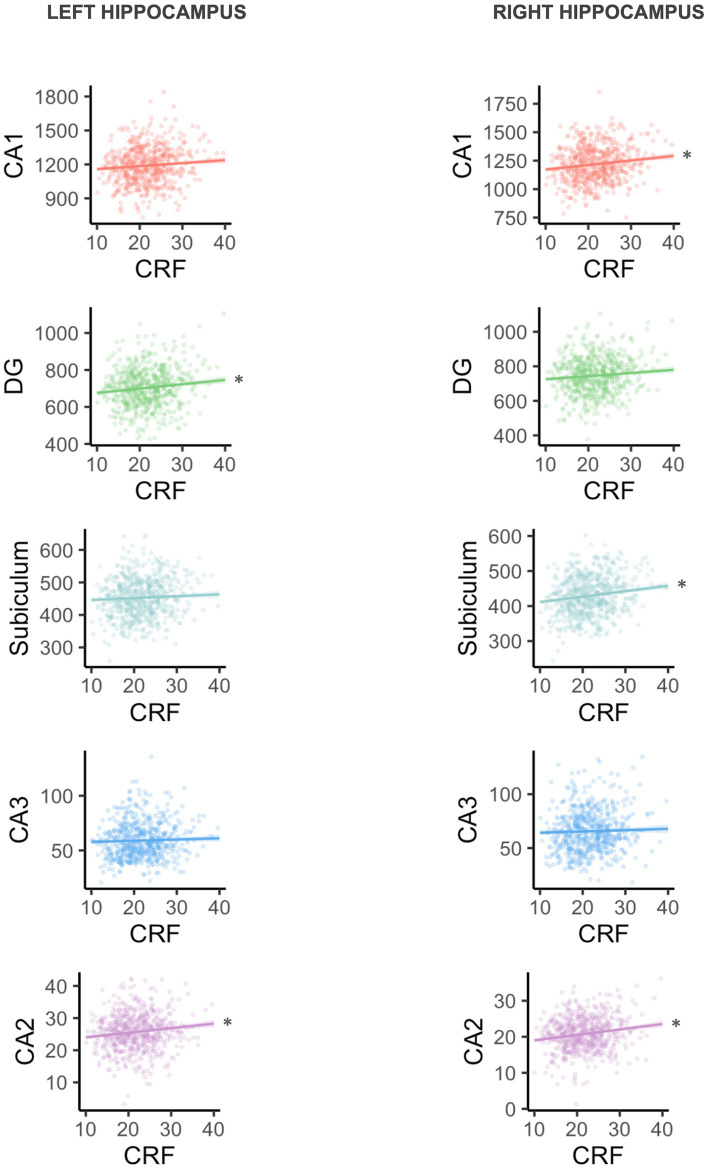
Plots are arranged in order of subfield size (CA1 is largest, CA2 is smallest). Volume is shown in mm^3^, and CRF is VO_2peak_ in ml/kg/min. *Statistically significant at uncorrected *p* < 0.05 and FDR < 0.2.

Separate linear regression models examined the association between CRF and left and right total hippocampal volume, left and right CA1 volume, left and right CA2 volume, left and right CA3 volume, left and right DG volume, and left and right subiculum volume. Results are presented in Table 3. Broadly, all regression coefficients were positive, such that higher CRF was associated with greater hippocampal subfield volumes. Higher CRF was significantly associated with greater total left (*B* = 5.82, *p* = 0.039) and total right hippocampal volume (*B* = 7.64, *p* = 0.006). However, consistent with our hypotheses, we found that the associations were only significant for certain subfields. For the left hippocampus, higher CRF was significantly associated with greater CA2 volume (*B* = 0.14, *p* = 0.022) and DG volume (*B* = 2.34, *p* = 0.031). For the right hippocampus, higher CRF was significantly associated with greater CA1 volume (*B* = 3.99, *p* = 0.011), CA2 volume (*B* = 0.15, *p* = 0.002), and subiculum volume (*B* = 1.56, *p* = 0.004) (Figure 1). These results remained statistically significant after correction for multiple comparisons.

### CRF × age interactions

**Figure 2:**
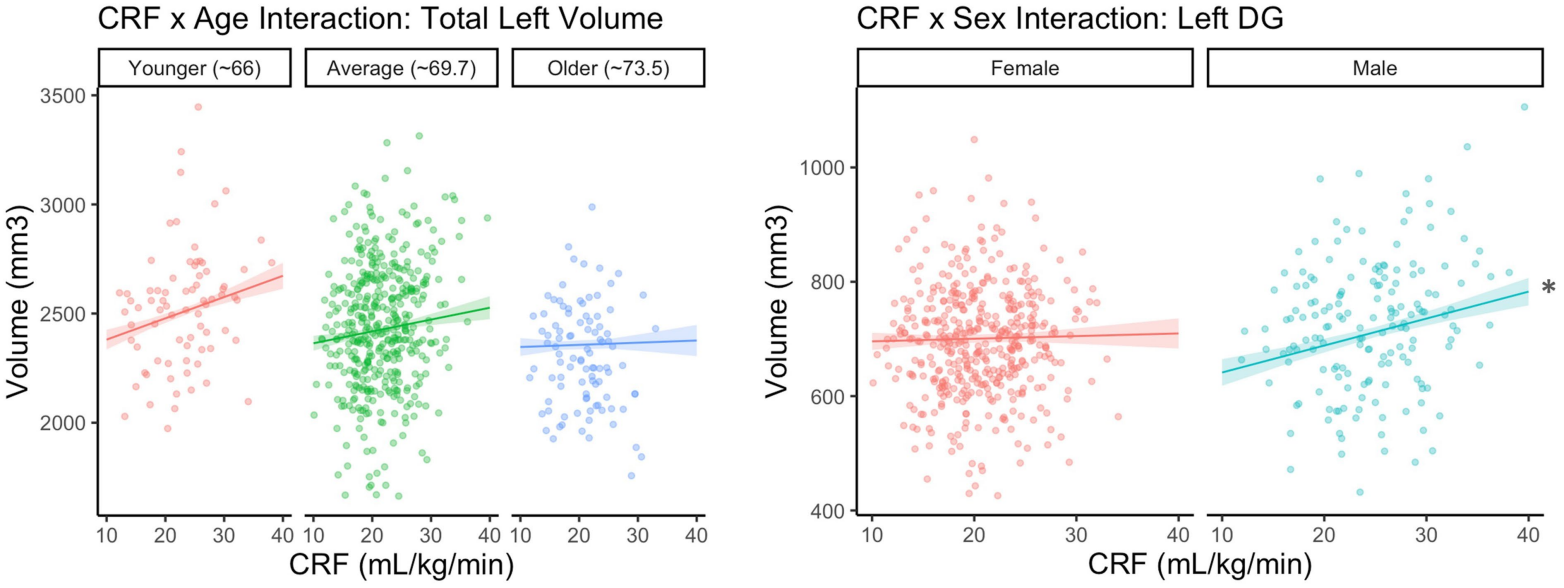
CRF × Age interaction is not significant after correction for multiple corrections. *Statistically significant at uncorrected *p* < 0.05 and FDR < 0.2.

Before correction for multiple comparisons, there was one significant CRF × age interaction for total left hippocampal volume (*B* = −1.17*, p* = 0.033). The simple slopes are depicted in Figure 2, such that the association was positive and statistically significant for those at the younger end of the age spectrum (−1 SD or ~ 66 years; estimate = 9.76, SE = 3.35, *p* = 0.004), but not statistically significant for those at the older end of the age spectrum (+1 SD or ~ 73.5; estimate = 1.05, SE = 3.58, *p* = 0.769) in this sample. However, the interaction did not meet criteria for statistical significance after correction for multiple comparisons and should be interpreted with caution. No other interactions with age were statistically significant.

### CRF × sex interactions

There was a statistically significant CRF × sex interaction for left DG volume (*B* = −4.26, *p* = 0.017), which remained after correcting for multiple comparisons. The simple slopes are depicted in Figure 2, such that the association between CRF and DG volume was positive and statistically significant for males (estimate = 4.72, SE = 1.46, *p* = 0.001), but not for females (estimate = 0.46, SE = 1.34, *p* = 0.731). No other interactions with sex were statistically significant.

Given the lower range of CRF in females (10.1–34.1 mL/kg/min) compared to males (11.9–39.6 mL/kg/min) in our sample, we conducted a sensitivity analysis removing the seven males with a CRF greater than 34.1 mL/kg/min. With these participants removed, the CRF × sex interaction for left DG volume was trending but no longer statistically significant (*B* = −3.24, *p* = 0.088).

### Association between CRF and episodic memory

In replication of the findings with the full baseline sample (*N* = 648) reported by Oberlin et al. (in press), the association between CRF and episodic memory was positive and statistically significant, such that higher CRF was associated with better episodic memory performance (*B* = 0.018, *p* = 0.005).

### Mediation analyses

**Figure 3:**
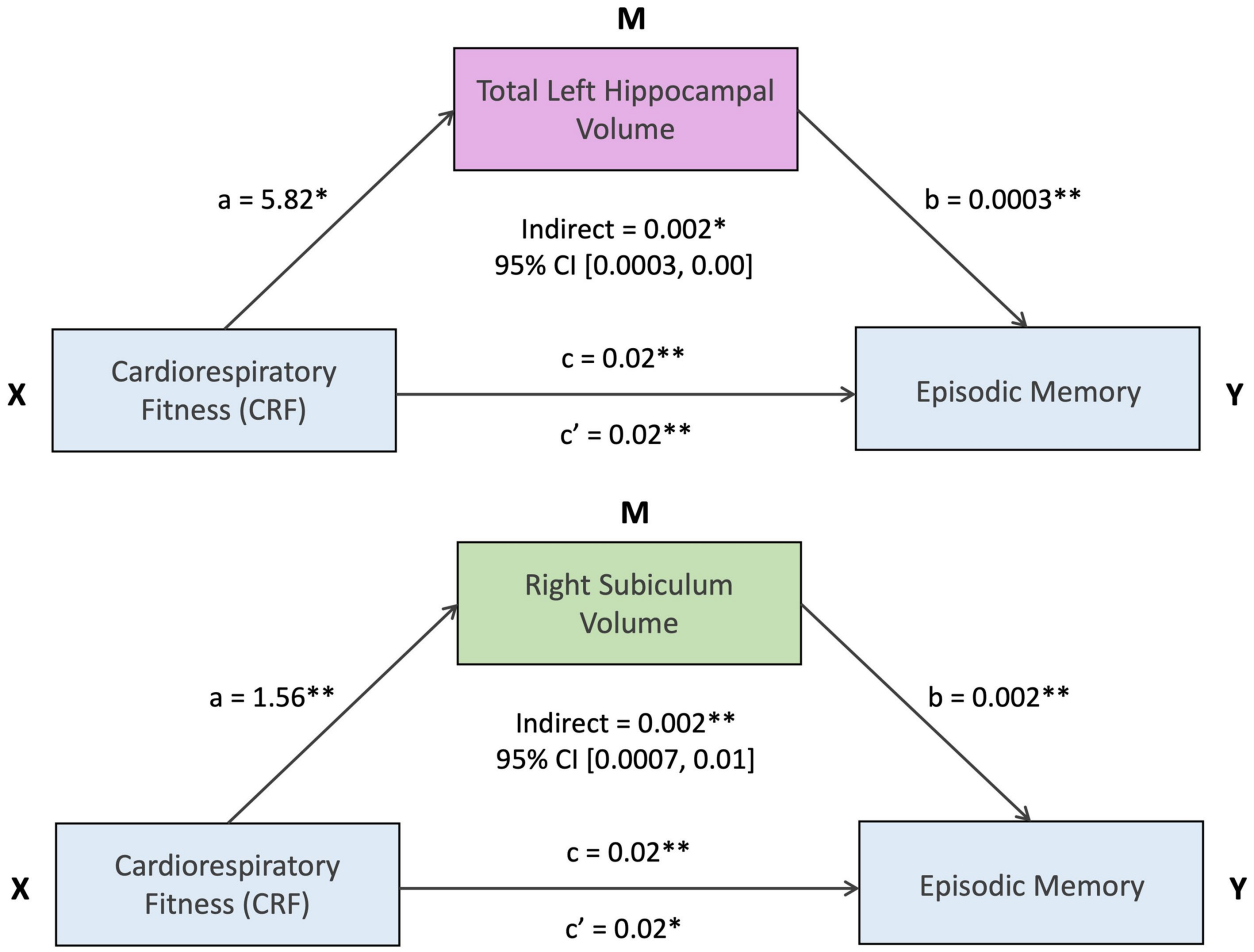
*Statistically significant at uncorrected *p* < 0.05 and FDR < 0.2. **Statistically significant at uncorrected *p* < 0.01 and FDR < 0.2.

Mediation analyses examined whether hippocampal volumes statistically mediated the association between CRF and episodic memory. Bootstrap analyses (bias-corrected and accelerated method) based on 5,000 resamples indicated statistically significant mediation for total left hippocampal volume [indirect effect = 0.002, 95% CI (0.0003, 0.00), *p* = 0.028], and right subiculum volume [indirect effect = 0.002, 95% CI (0.0007, 0.01), *p* = 0.006]. (Figure 3). These results remained significant after correction for multiple comparisons. No other hippocampal volumes were significant mediators.

## Discussion

Consistent with our predictions, we demonstrated that higher CRF was significantly associated with larger left and right total hippocampal volume, but that these associations were specific to certain subfields of the hippocampus. For the left hippocampus, higher CRF was significantly associated with larger CA2 and DG volumes. For the right hippocampus, higher CRF was significantly associated with larger CA1, CA2, and subiculum volumes. Further, we found that sex moderated the association between CRF and left DG volume, such that the association was positive and statistically significant for males, but not for females. Finally, we demonstrated that total left hippocampal volume and right subiculum volume statistically mediated the association between CRF and episodic memory performance.

Higher CRF has been previously associated with larger total hippocampal volume (Bugg et al., 2012; Erickson et al., 2009; Fletcher et al., 2016; McAuley et al., 2011; Szabo et al., 2011), but some studies have failed to find an association (Cole et al., 2020; Dougherty et al., 2017; Engeroff et al., 2018; Honea et al., 2009; Niemann et al., 2014; Raichlen et al., 2020). We hypothesized that this variability between studies might be explained by underlying heterogeneity within the hippocampal structure, such that only some subfields are associated with CRF. Our results support our hypothesis: only half of the subfields we examined were significantly associated with CRF. Despite this regional specificity, total hippocampal volume was still significantly associated with CRF in our sample. However, our results suggest that a non-significant association with total hippocampal volume in prior studies may be masking significant associations with underlying subfield volumes. Thus, examining hippocampal subfield volumes may offer more precision when characterizing the relationship between CRF and hippocampal morphology, and future studies might benefit from including subfield volumes in their analyses.

Our study demonstrated that higher CRF was significantly associated with larger right CA1 and subiculum volume, providing some support for the idea that CRF might help preserve the same subfields which are especially vulnerable to AD-related atrophy (Small et al., 2011; de Flores, De Flores et al., 2015). These results are consistent with studies demonstrating that smaller hippocampal volume is predictive of AD (McRae-McKee et al., 2019), and that higher CRF is associated with a reduced risk of AD (Tari et al., 2019). It will be important for future studies to examine whether the reduction in AD risk with higher CRF is mediated by variation in CA1 and subiculum volumes. Our results demonstrated that higher CRF was significantly associated with larger left DG volume. This aligns with one study of young adults (aged 18–35) found that higher CRF was associated with greater combined CA3 and DG volume, and that CA3/DG volume increased after a three-month aerobic exercise intervention (Nauer et al., 2020). Whether relationships among changes in CRF and hippocampal subfields are observed across the one-year IGNITE intervention remains to be determined.

It is important to speculate about the possible mechanisms underlying these associations between CRF and subfield volume. Studies in rodents indicate that exercise-induced change to hippocampal morphology is likely driven by increased secretion of neurotrophic substances that promote neurogenesis [e.g., Brain-Derived Neurotrophic Factor (BDNF)], angiogenesis (i.e., increase in vascularization), and increased synaptic plasticity, or synaptogenesis (i.e., increased ability to create new connections between neurons) (Ferrer-Uris et al., 2022). This literature is mirrored by histological studies in rodents (Kuhn et al., 1996; Seib and Martin-Villalba, 2014; Ingraham et al., 2008) and humans (Boldrini et al., 2018; Moreno-Jiménez et al., 2019) indicating that aging results in decreased neurogenesis and angiogenesis in the hippocampus. Although our results in this study are cross-sectional, and CRF is distinct from exercise, it might be that the physical activity required to maintain higher levels of CRF in older adulthood is neuroprotective and helps counteract the effects of aging on hippocampal morphology.

Age and sex could be important moderators of the association between CRF and hippocampal volume (Barha et al., 2019; Barha and Liu-Ambrose, 2018; Colcombe et al., 2003). We found that age moderated the association between CRF and total left hippocampal volume in uncorrected models, though this was no longer significant after adjusting for multiple comparisons. There was a narrower range of CRF in the older adults in our sample (see Supplementary Table 2), so it is possible that we were underpowered to detect an association between CRF and total left hippocampal volume in the oldest adults because of that restricted range. We found that sex moderated the association between CRF and left DG volume, such that the association was positive and significant for males but not females. Prior studies have found sex differences in the expression of BDNF as a result of aerobic exercise, with some studies reporting that males experience greater exercise-related increases in BDNF (Szuhany et al., 2015), while others have found the opposite (Barha et al., 2017). Countless studies report sex differences in the relationship between CRF or exercise and brain health, yet few are able to pinpoint specific mechanisms (Barha et al., 2019; Barha and Liu-Ambrose, 2018). In our sample, since males were fitter on average and had a wider range of CRF, we conducted a sensitivity analysis removing the males with a CRF that was higher than any of the females. These results indicated that the CRF × sex interaction for left DG volume was driven by males with higher CRF. Given the narrower range of CRF in the females in our sample, we may have been underpowered to detect a significant association between CRF and left DG volume in females. It will be important to examine sex differences in the context of the intervention, where we will test whether increasing CRF will increase hippocampal subfield volumes, and whether the effects differ between males and females.

We also demonstrated that the associations between CRF and hippocampal volume were behaviorally relevant. That is, total left hippocampal volume and right subiculum volume statistically mediated the relationship between CRF and episodic memory performance. These results suggest that CRF-related variation in hippocampal volume could be a possible mechanism by which CRF relates to episodic memory performance in late adulthood. Furthermore, the fact that there was some regional-specificity of these mediating patterns suggests that associations with episodic memory are relatively specific, and that the behavioral relevance of the hippocampal associations needs to be further examined in other studies.

We also report some hemispheric differences in our associations. This supports numerous prior studies reporting that higher CRF is associated with larger volume of the left hippocampus but not the right (Aghjayan et al., 2020; Esteban-Cornejo et al., 2017; Makizako et al., 2013; Nauer et al., 2020; Stillman et al., 2018). Several studies have also documented aging- and AD-related asymmetry in the volume of the hippocampus, where the left hippocampus tends to be more vulnerable and associated with cognitive impairment (Ardekani et al., 2019; de Toledo-Morrell et al., 2000; Shi et al., 2009; Vijayakumar and Vijayakumar, 2012). In our study, CRF was associated with both total left and right hippocampal volume, but the underlying subfield associations were different across hemispheres (left CA2 and DG, versus right CA1, CA2, and subiculum). In addition, our significant moderation effect was in the left hippocampus, and our mediation effects were significant for left total volume and right subiculum volume. Given that aging- and AD-related atrophy may be asymmetrical, it is plausible that CRF-related volume differences may be also, and that these CRF-associated differences in left versus right hippocampal volume may have clinical significance. It will be important to determine whether these hemispheric differences are maintained in the context of an exercise intervention.

Although our findings were statistically significant and associated with episodic memory performance, effect sizes were small. Nonetheless, in our standardized models the association of CRF with hippocampal volumes was approximately half the effect size of age—which is considered the most significant risk factor for hippocampal atrophy—indicating that even though the effect size for the association between CRF and hippocampal volumes is considered small, it could still be clinically meaningful. However, because other studies investigating CRF and total hippocampal volume have demonstrated larger effect sizes, it may be that our sample had several unique characteristics that diminished effect sizes of the associations. In particular, IGNITE excluded people that fell toward the cognitive impairment range while targeting physically inactive, yet healthy adults, which could have led to a sample in which the relationship between CRF and hippocampal volume was weaker than in other, more inclusive, studies.

Another possibility is our somewhat restricted range of CRF. Although we were able to detect significant associations between CRF and hippocampal volumes, our participants generally fell into the low-fit range and were sedentary. It is possible that with greater variability in CRF, the effect sizes for these associations would have been larger. However, another possibility is that the benefits to hippocampal volumes plateau at higher CRF levels and it may be that a loss of hippocampal volume is more related to lower CRF levels, as is represented in our sample. It is important to note that although we refer to lower and higher CRF, we use the term “higher CRF” as a relative term within our sample. In other words, the participants at the upper range of CRF were higher fit than those at the lower range, but they were not high-fit individuals relative to the population.

The results of the current study should be interpreted in the context of several limitations. The sample was highly educated, mostly female, and largely White, although it is important to note that the racial demographics matched that of the recruitment cities (Pittsburgh, Kansas City, and Boston). This was a cross-sectional study, so we cannot draw causal conclusions from these reported associations. It will be important to examine whether exercise interventions also show selective effects on certain hippocampal subfields, and whether those are the same subfields that are associated with CRF. There could be subtle variations in subfield segmentation that depend on the segmentation algorithms that were used and which are always evolving and improving. In particular, automated segmentation algorithms may be less accurate at delineating subfields within the hippocampal head and tail due to the greater anatomical complexity of those areas compared to the hippocampal body (Yushkevich et al., 2015). In addition, a T2-weighted TSE sequence provides high in-plane resolution, but the through-plane resolution is relatively coarse, potentially resulting in some blurred edges along the long-axis of the hippocampus. Nonetheless, we visually examined each participant’s segmentation for accuracy, and the only segmentations that were inaccurate—and therefore excluded from analyses—were those with only a partial hippocampal image due to incorrect placement of the field of view during the scan. Thus, we can be confident of the accuracy of the segmentations reported here. Future research should continue to examine the association between CRF and hippocampal subfields as new MRI acquisition protocols, segmentation algorithms, and quality assurance procedures are validated (Canada et al., 2023; Wisse et al., 2017).

Despite these limitations, the current study had a number of important strengths. We used a high-resolution T2-weighted focal hippocampal sequence, which allowed us to reliably segment the hippocampus into its subfields in a sample (*N* = 601) that is several times larger than previous studies examining relationships between CRF and hippocampal volume. Participants all underwent a graded exercise test with objective measurement of VO_2peak_, which is the gold standard for CRF assessment and is not commonly measured in studies of this size. The composite measure of episodic memory performance was derived from outcomes of six different neuropsychological tests, providing higher reliability than outcome measures from a single assessment, and was validated with a confirmatory factor analysis (Oberlin et al., in press). The quality of these assessments and large sample size allowed us to clarify the relationships among CRF, hippocampal subfield volumes, and episodic memory performance in healthy older adults.

## Conclusion

This study demonstrated that higher CRF was significantly associated with total left and total right hippocampal volume, but that these associations were specific to the left CA2 and DG, and the right CA1, CA2, and subiculum. These novel findings suggest that associations with CRF are regionally-specific within the hippocampus, and that total hippocampal volume might be an incomplete metric for characterizing the relationship between CRF and hippocampal morphology. Further, we demonstrated that sex was a significant moderator, and that our CRF-hippocampal volume findings have clear implications for episodic memory performance. Finally, our findings provide the first evidence that the associations between CRF and hippocampal subfields may be asymmetrical (i.e., the associations differ by hemisphere). We conclude that hippocampal measurement precision—by segmenting the hippocampus into its subfields, and keeping left and right hippocampi separate—is important for clearly interrogating the associations between CRF and hippocampal morphology. Collectively, these results provide the most precise and definitive evidence to date that the association between CRF and hippocampal volume in older adults is regionally-specific, moderated by sex, and has clinical relevance for episodic memory.

## Data Availability

The raw data supporting the conclusions of this article will be made available by the authors, without undue reservation.
