# Supplementary material for: Cardiorespiratory fitness, hippocampal subfield morphology, and episodic memory in older adults
Source: Front Aging Neurosci. 2024 Dec 19;16:1466328. doi: 10.3389/fnagi.2024.1466328 (PMC11694150; doi:10.3389/fnagi.2024.1466328)
Supplement: Supplementary file 1 [file Table_1.DOCX]

**Supplemental Table 1**

*Participant Characteristics Stratified by Sex*

| **Measure** | **Women**  (n = 434) | **Men**  (n = 167) |
| --- | --- | --- |
| Age (mean, SD) | 69.4 (3.6)* | 70.5 (4.0) |
| Race (n, %) |  |  |
| Caucasian/White | 316 (72.8)* | 143 (85.6) |
| African American/Black | 98 (22.6)* | 15 (9.0) |
| Asian | 7 (1.6) | 2 (1.2) |
| Other | 7 (1.6) | 2 (1.2) |
| Bi-racial | 6 (1.4) | 4 (2.4) |
| Native Hawaiian or other Pacific Islander | 0 | 1 (0.6) |
| Years of Education (mean, SD) | 16.1 (2.2)* | 16.8 (2.2) |
| BMI (kg/m^2^) (mean, SD) | 29.8 (6.0) | 29.9 (4.8) |
| CRF (VO_2peak_, ml/kg/min) (mean, SD) | 20.6 (4.5)* | 24.48 (5.5) |
| CRF Range (min – max) | 10.10 – 34.1 | 11.90 – 39.6 |
|  |  |  |

*Significantly different between groups (*p* < 0.05)
