# Supplementary material for: Cardiorespiratory fitness, hippocampal subfield morphology, and episodic memory in older adults
Source: Front Aging Neurosci. 2024 Dec 19;16:1466328. doi: 10.3389/fnagi.2024.1466328 (PMC11694150; doi:10.3389/fnagi.2024.1466328)
Supplement: Supplementary file 2 [file Table_2.DOCX]

**Supplemental Table 2**

*Participant Characteristics Stratified by Age*

| **Measure** | **Younger**  (n = 76) | **Average**  (n = 428) | | **Older**  (n = 97) | |
| --- | --- | --- | --- | --- | --- |
| Age (mean, SD) | 65 (0) | 69.1 (2.2) | | 76.2 (1.8) | |
| Race (n, %) |  |  | |  | |
| Caucasian/White | 55 (72.4) | 331 (77.3) | | 73 (75.3) | |
| African American/Black | 16 (21.1) | 77 (18) | | 20 (20.6) | |
| Asian | 2 (2.6) | 6 (1.4) | | 1 (1) | |
| Other | 0 | 6 (1.4) | | 3 (3.1) | |
| Bi-racial | 3 (3.9) | 7 (1.6) | | 0 | |
| Native Hawaiian or other Pacific Islander | 0 | 1 (0.2) | | 0 | |
| Years of Education (mean, SD) | 16.2 (2.2) | 16.3 (2.3) | | 16.4 (2.3) | |
| BMI (kg/m^2^) (mean, SD) | 30.1 (5.9) | 30.1 (5.8) | | 28.5 (4.7) | |
| CRF (VO_2peak_, ml/kg/min) (mean, SD) | 23.2 (5.8) | 21.6 (5) | | 20.8 (4.6) | |
| CRF Range (min – max) | 12.2 – 38.1 | 10.1 – 39.6 | | 11.60 – 33.0 | |
|  |  | |  | |  |

Younger = participants whose age is at least 1 SD below the sample mean (< 66)

Average = participants whose age is between 1 SD below and above the sample mean (> 66 and < 73.5)

Older = participants whose age is at least 1 SD above the sample mean (> 73.5)
